# Identification of *Shaker* Potassium Channel Family Members in *Gossypium hirsutum* L. and Characterization of *GhKAT1aD*

**DOI:** 10.3390/life13071461

**Published:** 2023-06-28

**Authors:** Qianqian Wang, Shuying Li, Fangjun Li, Xiaoli Tian, Zhaohu Li

**Affiliations:** State Key Laboratory of Plant Physiology and Biochemistry, College of Agronomy and Biotechnology, China Agricultural University, No. 2 Yuanmingyuan Xi Lu, Haidian District, Beijing 100193, China; wangqianqian@cau.edu.cn (Q.W.);

**Keywords:** cotton, potassium, *Shaker* K^+^ channel gene family, gene function

## Abstract

K^+^ channels of the *Shaker* family have been shown to play crucial roles in K^+^ uptake and transport. Cotton (*Gossypium hirsutum*) is an important cash crop. In this study, the 24 *Shaker* family genes were identified in cotton. Phylogenetic analysis suggests that they were assigned to five clusters. Additionally, their chromosomal location, conserved motifs and gene structure were analyzed. The promoter of cotton *Shaker* K^+^ channel genes comprises drought-, low-temperature-, phytohormone-response elements, etc. As indicated by qRT-PCR (quantitative real-time PCR), cotton *Shaker* K^+^ channel genes responded to low K^+^ and NaCl, and especially dehydration stress, at the transcript level. Moreover, one of the *Shaker* K^+^ channel genes, *GhKAT1aD*, was characterized. This gene is localized in the plasma membrane and is predicted to contain six transmembrane segments. It restored the growth of the yeast mutant strain defective in K^+^ uptake, and silencing *GhKAT1a* via VIGS (virus-induced gene silencing) resulted in more severe symptoms of K^+^ deficiency in cotton leaves as well as a lower net K^+^ uptake rate. The results of this study showed the overall picture of the cotton *Shaker* K^+^ channel family regarding bioinformatics as well as the function of one of its members, which provide clues for future investigations of cotton K^+^ transport and molecular insights for breeding K^+^-efficient cotton varieties.

## 1. Introduction

Potassium (K^+^), the most abundant cation in plant cells, is of essential importance to the growth and development of plants at all stages and determines the final crop yield and quality [1]. For example, it is a key player in the activation of enzymes, neutralization of negative charges on proteins, cellular turgor and elongation, stomata and leaf movements, translocation of photosynthates, etc. [2]. Typically, the K^+^ concentration ranges between 100 and 200 mM in the cytoplasm [3]. In contrast, the K^+^ concentration in soil solution is only 10–100 μM [4]. K^+^ is taken up from the soil by root epidermal and cortical cells. Once K^+^ is inside the root symplast, it may be stored in vacuoles or transported to the shoot via the xylem [5]. In turn, shoot cells may also supply stored K^+^ for redistribution via the phloem. In this transit from the soil to the different plant organs, K^+^ crosses various cell membranes through K^+^-specific transport systems, including K^+^ channel proteins and K^+^ transporter proteins [6].

K^+^ channels are present in the plasma membrane, or tonoplast, of plant cells. They can be classified into three categories: *Shaker*-like voltage-dependent, tandem-pore (TPK) and two-pore channels (TPC) [7]. The *Shaker* K^+^ channels have been confirmed as the critical functional proteins for K^+^ absorption and transport. A complete *Shaker* K^+^ channel comprises four subunits, each with six transmembrane domains (TMS). Nine *Shaker* K^+^ channel gene family members have been identified in *Arabidopsis* [8], and they fall into five clusters according to the amino acid sequence similarity, as well as three categories regarding their response to the membrane voltage [9]. The first category is inwardly rectifying K^+^ channels, activated primarily at hyperpolarized membrane potentials and responsible for K^+^ absorption. AKT1 belongs to this category and is the first characterized K^+^ channel in *Arabidopsis*. Recently, Lu et al. (2022) reported a regulatory mechanism of AKT1 activity through conformational changes associated with symmetric rearrangement [10]. The second category refers to the outward rectifying K^+^ channel, which should be activated when the membrane potential is depolarized, mainly involved in the extracellular release of K^+^. The third is weakly rectifying K^+^ channels, which play a certain role in K^+^ influx and efflux. In addition, as a member of the *shaker* K^+^ channel gene family, *KAT3* (*KC1*) does not have K^+^ absorption or transport functions and should interact with inward rectifier channels (e.g., *AtAKT1*) to form heteromorphic K^+^ channels, thus regulating activity and preventing K^+^ leakage in root cells under low K^+^ conditions [11].

K^+^ transporters in plants comprise KUP/HAK/KT, HKT (high-affinity K^+^ transporter) and CPA (cation/hydrogen exchanger) transporter families [12]. The KUP (K^+^ uptake permease) transporter has 10–14 transmembrane domains and a long loop between the second and third TMS [13]. The HKT transporter covers eight transmembrane domains with a pore–loop structure between each two transmembrane domains [14]. CPA refers to a larger family in plants, with monovalent cation/proton anti-transport functions, containing two sub-CPA1 and CPA2 [15].

Cotton (*Gossypium hirsutum* L.) is a K^+^-loving crop and requires considerable K^+^ during its growth and development. Nearly 50% of K^+^ in cotton plants is located in bolls and 24% in seeds and lint [16]. Under K^+^-deficiency, cotton leaves first exhibit chlorosis between the veins, and then chlorosis manifests as yellow and brown spots. Furthermore, the leaf tip and leaf edge curl down. Lastly, the whole leaves turn rust-colored, become brittle, and then fall off prematurely [17,18]. As a result, the plant height, leaf area, dry matter and boll mass are decreased, and the ability of cotton plants to resist biotic and abiotic stresses declines [19,20,21,22].

In contrast to the importance of K^+^ for cotton, there has been limited information available on the molecular mechanisms of cotton K^+^ uptake and transport. Up until now, only *GhAKT1* and *GhAKT2* in the *Shaker* K^+^ channel gene family have been reported. *GhAKT1* encodes a plasma membrane-localized protein and can mediate K^+^ uptake in roots at 100 µM K^+^ [23]. The *GhAKT2bD* is expressed in xylem and phloem and facilitates K^+^ allocation in cotton plants [24]. Considering the crucial function of *Shaker* K^+^ channels, it is necessary to obtain a full view of the cotton *Shaker* K^+^ channel gene family members.

In this study, the 24 *Shaker* genes were identified from the genomes of cotton, and their distribution in the genome, the motif distribution and characterization of gene structure, and cis-elements in promoter regions were predicted. Then, the expression profiles of these members as well as their responses to K^+^ deficiency, salt and dehydration were examined. Moreover, the characterization and function of one of the *Shaker* K^+^ channel gene family members, *GhKAT1aD*, were revealed. The results provide a basis for the characteristics of cotton K^+^ channels as well as a molecular perspective for improving cotton K^+^ nutrient utilization efficiency.

## 2. Materials and Methods

### 2.1. Plant Materials and Treatments

In this study, the upland cotton cultivar R15 was employed. Seeds were surface sterilized after being soaked in 9% H_2_O_2_ for 30 min. Subsequently, the seeds were surface sterilized by soaking in 3% H_2_O_2_ for 30 min, then rinsed with tap water and soaked in deionized water overnight. For hydroponic culture, seeds were germinated in a K^+^-free sand medium for 3 days. Next, the seeds were transferred into plastic pots with a half-strength modified Hoagland’s solution containing 2.5 mM KNO_3_, 2.5 mM Ca(NO_3_)_2_, 1 mM MgSO_4_, 0.5 mM (NH_4_)H_2_PO_4_ and 0.1 mM FeNaEDTA, as well as 2 × 10^−4^ mM CuSO_4_, 1 × 10^−3^ mM ZnSO_4_, 2 × 10^−2^ mM H_3_BO_3_, 5 × 10^−6^ mM (NH_4_)_6_Mo_7_O_24_ and 1 × 10^−3^ mM MnSO_4_. In the process of cultivation, the medium was renewed every three days and continuously aerated with an air pump to provide O_2_. At the three-leaf stage, the seedlings were treated with K^+^ deficiency (30 μM K^+^) or salt stress (250 mM NaCl) and/or dehydration (15% PEG6000). The root tips and the third leaf with three biological replicates were collected at 0, 6, 12, 24 and 48 h after treatments. The samples were immediately frozen in liquid nitrogen and then stored at −80 °C.

### 2.2. Identification and Phylogenetic Analysis of Members of the Shaker K^+^ Channel Gene Family

The genome and protein sequences of *Gossypium hirsutum* were downloaded from CottonFGD (https://cottonfgd.org/about/download.html, accessed on 9 July 2021). The data for *Arabidopsis thaliana* and rice were downloaded from TAIR (https://www.arabidopsis.org/, accessed on 9 July 2021) and the China Rice Data Center (https://ricedata.cn, accessed on 9 July 2021), respectively. The *Arabidopsis Shaker* genes served as the query sequence, and Blastp was employed to search with the threshold E < 1 × 10^−200^ so as to obtain more complete members of the *Shaker* K^+^ channel gene family. Subsequently, the results from HMM and Blastp were fused, and the CDD database at NCBI online (https://www.ncbi.nlm.nih.gov/, accessed on 9 July 2021) was employed to retain members with correct and complete domains.

With the use of Clustal W of MEGA 7.0 software (https://www.megasoftware.net/, accessed on 9 July 2021), the amino acid sequences of *Shaker* family members of *Arabidopsis*, rice and cotton were aligned, and the phylogenetic tree was established using the neighbor-joining method. Analyses with 1000 replicates were conducted to evaluate the tree structure. Phylogenetic trees were beautified using the online tool Evolview V3 (https://www.evolgenius.info//evolview/#login, accessed on 9 July 2021).

### 2.3. Analysis of Motifs and Gene Structures

The target protein sequence was integrated into a text file, the sequence was kept in the FASTA format, and the online tool MEME (http://alternate.memesuite.org/tools/meme, accessed on 18 July 2021) was adopted to search for motifs. The gene structure was displayed using Tbtools (https://github.com/CJ-Chen/TBtools/releases, accessed on 18 July 2021) based on introns and exons in gene annotation files.

### 2.4. Chromosomal Localization of the Shaker K^+^ Channel Gene Family

The location of the *Shaker* K^+^ channel gene family on the chromosome was identified using a gene annotation file (GFF). Subsequently, the location map was generated using Mapchart 2.32 software (https://www.mapchart.net, accessed on 14 July 2021).

### 2.5. Analysis of Cis-Regulatory Elements

Cis-acting element predictions were performed using the PlantCare website (http://bioinformatics.psb.ugent.be/webtools/plantcare/html/, accessed on 13 July 2021), and the results were collated and then simplified. The results were mapped using TBtools v1.098667 software (https://github.com/CJ-Chen/TBtools/releases, accessed on 13 July 2021).

### 2.6. Gene Expression Profile and Their Responses to Stress Conditions

The raw transcriptome data of roots, stems, leaves, floral organs (e.g., petals, receptacles, calyx, bracts, anthers, filaments and pistils), ovule fiber mixture (3~5 DPA (Days post-anthesis)), ovules (10~25 DPA) and fibers (10~25 DPA) of the upland cotton genetic standard line TM-1 were downloaded from NCBI SRA database (PRJNA490626) (https://www.ncbi.nlm.nih.gov/sra, accessed on 9 July 2021). The original reads were quality-controlled using Cutadapt 2.6 software to remove sequencing adapter sequences (https://github.com/marcelm/cutadapt/, accessed on 9 July 2021). The reads of all samples were aligned to the genome of *Gossypium hirsutum* ‘TM-1’ NAU using HISAT2 T 2.1.0 software, and the reads on the unique alignment were screened to provide a basis for downstream analysis (https://www.psc.edu/resources/software/hisat-2/, accessed on 9 July 2021). The assembly and quantification of transcripts were performed using StringTie (https://ccb.jhu.edu/software/stringtie/index.shtml, accessed on 9 July 2021), and the FPKM (fragments per kilobase of exon model per million mapped fragments) value served as the normalized expression level.

Furthermore, our previous three groups of the root transcriptome data (1 cm radicle tip at the cotyledonary stage of Acala1517-08, 2 cm lateral roots at the three-leaf stage of Xinshi 17, and 5 cm lateral roots at the three-leaf stage of Lumianyan 22) and three groups of leaf transcriptome data of upland cotton (the young leaf at the two-leaf stage of Lumianyan 22, the young leaf at the three-leaf stage of Lumianyan 22 and the fourth leaf at the six-leaf stage of Xinshi 17) were studied. After log2 normalization of FPKM values, heat maps of gene expression were drawn using TBtools software.

### 2.7. Quantitative Real-Time PCR (qRT-PCR) Analysis of Shaker Genes

The total RNA was extracted using a TIANGEN RNA extraction kit (TransGen Biotech, Beijing, China) according to the manufacturer’s instructions. The cDNA was synthesized using the PrimeScriptTM RT Reagent Kit with gDNA Eraser (Takara, Dalian, China). The primer sequence is shown in Supplementary Table S1. The qRT-PCR analysis was carried out using iTaq SYBR green Supermix (Bio-Rad, Thermo Fisher Scientific, Shanghai, China) with an ABI 7500 Real-Time PCR system (Applied Biosystems, Waltham, MA, USA) with three technical replicates of each sample under the following conditions: 95 °C for 30 s, followed by 40 cycles of 95 °C for 5 s and 60 °C for 34 s, with a final extension at 60 °C for 1 min. The expression level of the respective gene was determined relative to *GhACTIN9* as a reference gene, and it was calculated using the 2^−△△CT^ method.

### 2.8. Subcellular Localization of K^+^ Channel GhKAT1aD

The coding sequence (CDS) of *GhKAT1aD* was amplified using the primers listed in Supplementary Table S1, subsequently inserted into the vector p*HBT::GFP* to obtain the *GhKAT1aD::GFP* fusion construct. This fusion construct and the cytomembrane marker p35S::PIP2-RFP were transferred into protoplasts prepared from cotton cotyledons and incubated under low light intensity (50 μmol m^−2^ s^−1^) at room temperature (25 °C) for 18 h. A Zeiss LSM900 (Carl Zeiss, Oberkochen, Germany) confocal laser scanning microscope was used to detect subcellular localization of the target protein.

### 2.9. Agrobacterium-Mediated Virus-Induced Gene Silencing (VIGS) of GhKAT1aD

Cotton VIGS assay was performed according to a previously described procedure [25]. A fragment of *GhKAT1aD* was amplified and inserted into the tobacco rattle virus (TRV) binary vector pYL156 between the *Eco*RI and *Kpn*I sites. Plasmids of pTRV-*RNA1* or pTRV-*RNA2* (pYL156-Ctrl, pYL156-*CLA1* (*cloroplastos alterados 1*) and pYL156-*GhKAT1aD*) were introduced into *Agrobacterium tumefaciens* strain GV3101 (TsingKe BioTech, Beijing, China) by the freeze–thaw method [26]. Agrobacteria were incubated overnight at 28 °C in YEP medium (50 μg·mL^−1^ kanamycin, 25 μg·mL^−1^ gentamicin, 10 mM MES and 20 μM acetosyringone). Cells were pelleted and resuspended in infiltration buffer (10 mM MgCl_2_, 10 mM MES and 200 mM acetosy·ringone). The OD_600_ nm of pTRV-*GhKAT1aD*, pTRV-Ctrl, pTRV-*CLA1* and pTRV-*RNA1* were adjusted to 1.5. The cells were incubated at room temperature for 3 h. For creating VIGS-*GhKAT1aD*, VIGS-Ctrl and VIGS-*CLA1* lines, cell suspensions of pTRV-*GhKAT1aD*, pTRV-Ctrl and pTRV-*CLA1*, respectively, were mixed at a 1:1 ratio with pTRV-*RNA1*. The mixtures were infiltrated into two fully expanded cotyledons of R15 seedlings (1-week-old seedlings grown in solutions with 2.5 mM K^+^) using a needleless syringe. After infiltration, the plants were kept in the dark overnight. At least 18 plants were inoculated for each construct. The VIGS-*CLA1* plants were used as markers to monitor the silencing reliability. The young leaves of two-week-old VIGS plants were sampled for real-time PCR to check the interference efficiency.

Subsequently, parts of VIGS-*GhKAT1aD* and VIGS-Ctrl plants were transferred to the modified Hoagland’s solution with low K^+^ (30 μM) or sufficient K^+^ (2.5 mM) for three days to determine the net K^+^ uptake rate (see below). The rest of the plants were grown under 30 μM or 2.5 mM K^+^ for 33 d, then harvested for photographing as well as measurement of the plant height and biomass, chlorophyll and K^+^ content.

### 2.10. Determination of Net K^+^ Uptake Rate of VIGS-GhKAT1aD Plants

The above VIGS-*GhKAT1aD* and VIGS-Ctrl plants grown under low K^+^ or sufficient K^+^ for three days were transferred to solutions containing 0.1 mM K^+^. After 10 h, the plants were collected to measure the fresh weight of the roots. Then, the net K^+^ absorption rate (µg/g·h) was calculated as [(C1V1 − C2V2) × M]/(T × W). C1 and C2 represent the concentration (µM) of K^+^ in the solution before and after absorption; V1 and V2 represent the volume (L) of solution before and after K^+^ uptake; M is the relative atomic mass of K^+^; T represents the K^+^ absorption time (10 h); W represents the root fresh weight (g).

### 2.11. Statistical Analysis

Microsoft Excel 2010 (Microsoft Corp, Albuquerque, NM, USA) was used for data organization and figure construction. The general linear model procedure in SPSS 21.0 (SPSS Inc., Chicago, IL, USA) was used for analysis of variance (ANOVA). Mean values were compared using Duncan’s multiple comparison procedure at the 1% or 5% level of probability.

## 3. Results

### 3.1. Phylogenetic Analysis of Shaker K^+^ Channel Proteins

A phylogenetic tree was evaluated and established using 38 protein sequences, including 24 from *Gossypium hirsutum*, nine from *Arabidopsis* and six from *Oryza sativa*. Subsequently, according to the taxonomic criteria of *Arabidopsis* and *Oryza sativa*, the members were divided into five groups (Clusters I–V). Referring to the gene names of *Arabidopsis*, the cotton *Shaker* family genes are named based on homologous relationships, where a, b, c and d represent relative distances, and A and D represent subgenome.

**Figure 1:**
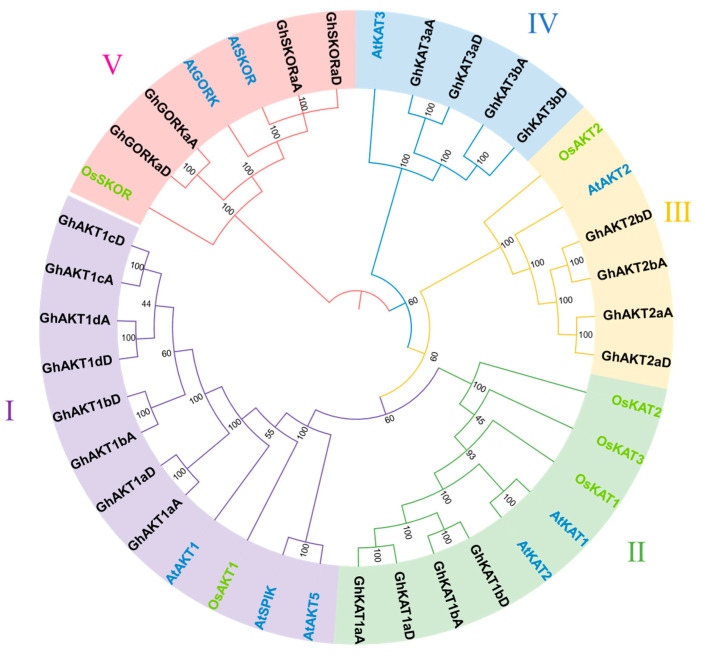
Phylogenetic tree of Shaker K^+^ channel proteins from *Arabidopsis*, rice (*Oryza sativa*) and cotton (*Gossypium hirsutum*). The numbers in the figure are bootstrap probability values.

As depicted in Figure 1, Cluster I had the most members, including eight GhAKT1s which accounted for 21.1% of the total members of the *Shaker* family. Clusters II, III and IV covered four GhKAT1s, four GhAKT2s and four GhKAT3s, respectively. The share of Cluster V was 10.5% and contained two GhSKORs and two GhGORKs.

### 3.2. Protein Motifs and Gene Structure Analysis of Shaker K^+^ Channel Family

**Figure 2:**
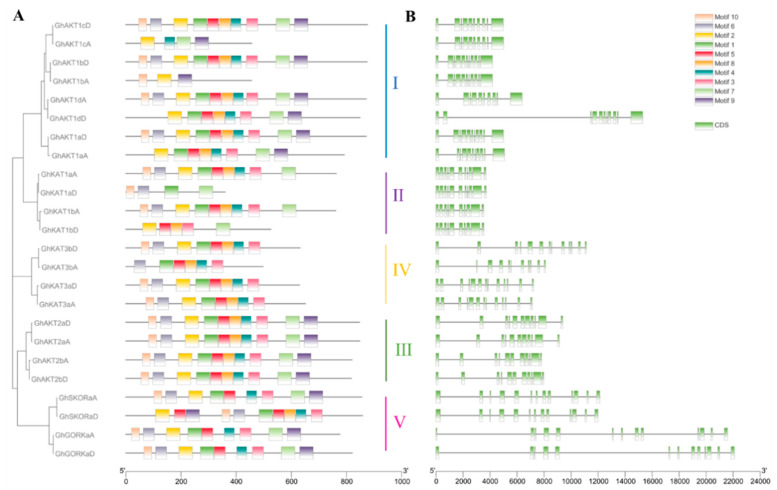
Motif analysis of protein (**A**) and gene CDS (coding sequence) structures (**B**) of the cotton *Shaker* K^+^ channel family.

The Shaker K^+^ channel gene family members of cotton contained up to 10 motifs named motif 1–10 (Figure 2A). There were three types of motif distributions among the members of Cluster I. To be specific, GhAKT1cD, GhAKT1bD, GhAKT1dA and GhAKT1aD covered motifs 1–10, presenting the same arrangement. GhAKT1dD and GhAKT1aA did not have motif 6 and motif 10, and the rest of the motifs were arranged similarly to the above GhAKT1s. GhAKT1cA and GhAKT1bA had only 3–4 motifs. In Cluster II, the members of the A subgenome (GhKAT1sA) contained nine motifs (without motif 9) and were arranged in the same way, whereas the members of the D subfamily (GhKAT1sD) only covered 4–5 motifs. In Cluster III, all members (GhAKT2s) involved motifs 1–10 and displayed the same arrangement. In Cluster IV, all members except GhKAT3bA involved eight motifs (without motifs 7 and 9) and displayed the same arrangement. Furthermore, in Cluster V, GhSKORaD comprised 10 motifs, and the other three genes had nine motifs (without motif 9) with the same arrangement.

The number of CDS (coding sequences) for the members of the entire *Shaker* family varied between 10 and 13, and the members of the same cluster generally exhibited a similar distribution of CDS (Figure 2B).

### 3.3. Chromosome Location of the Shaker K^+^ Channel Gene Family

**Figure 3:**
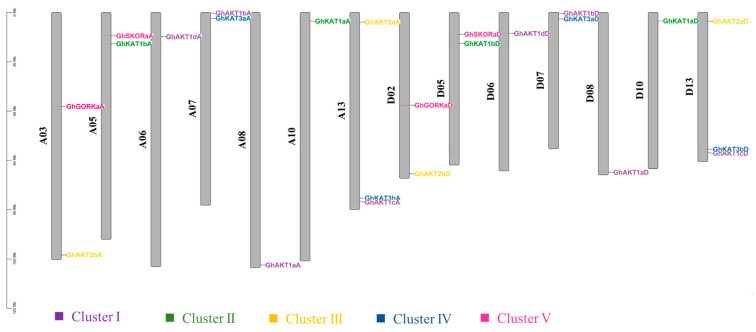
Chromosomal distributions of cotton *Shaker* K^+^ channel genes. The chromosome number is shown to the left of each vertical bar. Gene names are shown on the right. The *Shaker* genes in the same cluster are displayed in the same color. The length of a chromosome is expressed in Mb (megabases).

Figure 3 presents the chromosome length of upland cotton and the location of the *Shaker* genes on the chromosome. There were 14 out of the 26 chromosomes in upland cotton that comprised *Shaker* K^+^ channel gene family members and the A and D subgenomic chromosomes, each of which had 12 *Shaker* genes. Moreover, chromosomes A13 and D13 covered the largest number (three) of *Shaker* genes.

Specifically, the members of Cluster I were located on chromosomes A06, A07, A08, A13, D06, D07, D08 and D13, and those of Cluster II were distributed on chromosomes A05, A10, D05 and D10. For members of Clusters III, IV and V, they were identified on A03, A13, D02 and D13 chromosomes; A07, A13, D07 and D13 chromosomes; and A03, A05, D02 and D05 chromosomes, respectively.

### 3.4. Analysis of Cis-Regulatory Elements of Shaker K^+^ Channel Gene Family

**Figure 4:**
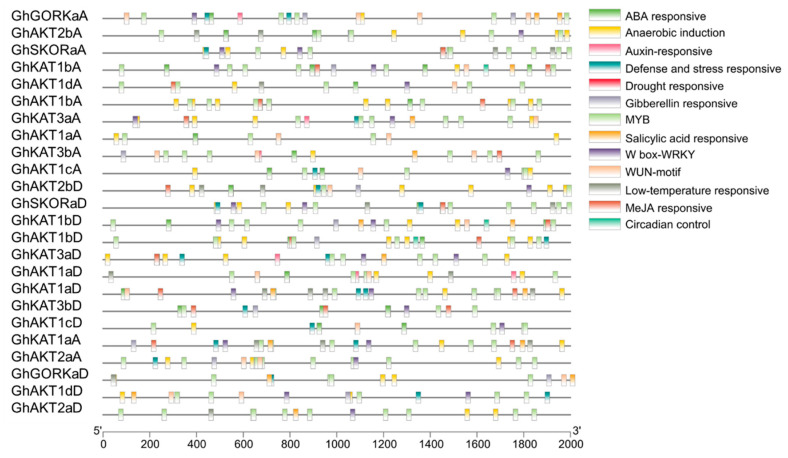
Analysis of cis-regulatory elements in the promoter regions of the cotton *Shaker* K^+^ channel gene family.

The cis-regulatory elements were predicted for the 2000 bp upstream of the coding region to clarify the possible functions of cotton *Shaker* K^+^ channel gene family (Figure 4). The main elements comprised the ABA response element, anaerobic induction, auxin response element, injury and stress response element, drought response element, gibberellin response element, salt damage response element, low temperature response element, MeJA response element, MYB, WRKY, WUN-motif and circadian control element. These results suggest that the expression of the *Shaker* K^+^ channel family may be regulated by a wide variety of phytohormones and abiotic stresses.

### 3.5. Gene Expression Pattern of Shaker K^+^ Channel Gene Family in Cotton

**Figure 5:**
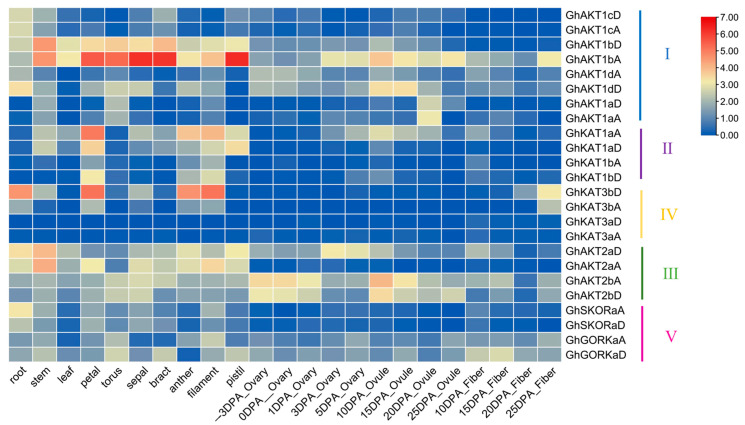
Expression levels of the cotton *Shaker* K^+^ channel gene family in different organs and tissues. The heat map was drawn after the FPKM values were normalized by log2 corresponding to each gene. DPA: days post-anthesis.

The publicly available RNA-seq database was employed to investigate the expression of *Shaker* genes in cotton roots, stems, leaves, floral organs (petals, receptacles, calyx, bracts, anthers, filaments and pistils), ovaries (−3~5 DPA), ovules (10~25 DPA) and fiber (10~25 DPA) (Figure 5). In Cluster I, all genes except *GhAKT1aA* and *GhAKT1aD* were highly expressed in roots. Furthermore, the expression levels of *GhAKT1bA* and *GhAKT1bD* were considerably higher in stem, leaf and flower organs than those in roots, and *GhAKT1bA* was also enriched in ovules and fibers during 3~25 DPA. Moreover, *GhAKT1dA* and *GhAKT1dD* were expressed at a certain level in the ovary at −3 and 0 DPA, and the latter was highly expressed in the ovule at 10 and 15 DPA.

All genes of Cluster II were lowly expressed in roots but highly expressed in the petals, anthers and filaments. Meanwhile, *GhKAT1aA* and *GhKAT1aD* were highly expressed in stems and leaves as well as pistils. The genes in Clusters III and V were expressed in nearly all parts of cotton plants, except *GhAKT2aA*, *GhSKORaA* and *GhSKORaD*, which were lowly expressed in the ovary (−3~5 DPA), ovule (10~25 DPA) and fiber (10~25 DPA). For the genes in Cluster IV, *GhKAT3aA* and *GhKAT3aD* were rarely expressed in all parts of cotton plants. Nevertheless, *GhKAT3bA* and *GhKAT3bD*, especially *GhKAT3bD*, were enriched in the root, stem and flower organs.

### 3.6. Expression of Cotton Shaker K^+^ Channel Gene Family in Response to Stress Conditions

The analysis of cis-elements in the promoter region indicated that the *Shaker* K^+^ channel gene family may play a certain role in the stress response. The upland cotton studied is formed by hybridization of A and D subgenome diploids, and these two subgenomes display comparable gene order and colinearity [27]. Therefore, the *Shaker* family genes from the D subgenome are chosen for qRT-PCR analysis considering the conservation of gene function.

**Figure 6:**
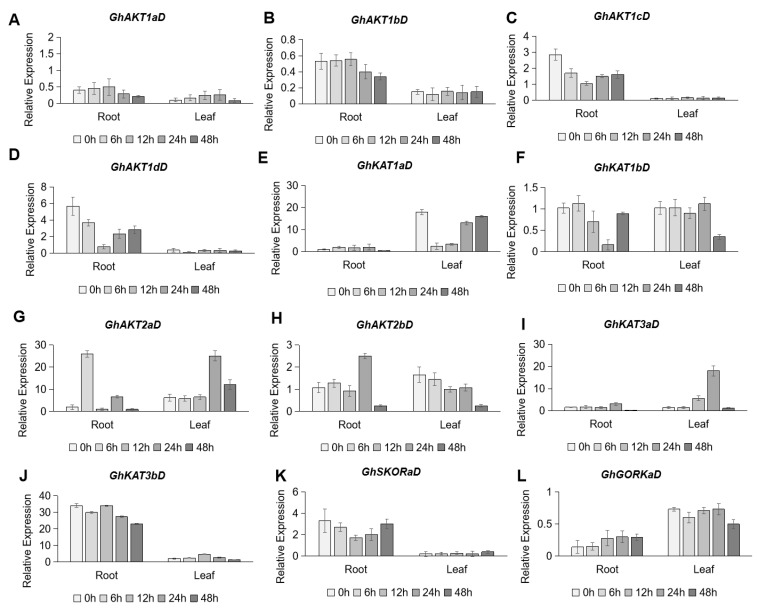
Time-course expression profiling of *Shaker* K^+^ channel family genes from the D subgenome in response to potassium deficiency stress (30 μM K^+^). (**A**–**L**) indicate *GhAKT1aD* (**A**), *GhAKT1bD* (**B**), *GhAKT1cD* (**C**), *GhAKT1dD* (**D**), *GhKAT1aD* (**E**), *GhKAT1bD* (**F**), *GhAKT2aD* (**G**), *GhAKT2bD* (**H**), *GhKAT3aD* (**I**), *GhKAT3bD* (**J**), *GhSKORaD* (**K**) and *GhGORKaD* (**L**), respectively.

As depicted in Figure 6, the *Shaker* family genes in cotton roots and leaves had no consistent responses to K^+^ deficiency (30 μM K^+^). In the roots, low K^+^ stress induced the down-regulation of *GhAKT1bD* (Figure 6B), *GhAKT1cD* (Figure 6C) and *GhAKT1dD* (Figure 6D) in Cluster I, and *GhKAT3bD* in Cluster IV (Figure 6J), but the up-regulation of *GhGKORaD* in Cluster V (Figure 6L). In the leaves, there were three *Shaker* K^+^ channel family genes, obviously responding to K^+^ deficiency. Among them, the expression of *GhKAT1aD* in Cluster II sharply decreased at 6 and 12 h after low K^+^ stress, and then drastically increased to the original level after 24 and 48 h of treatment (Figure 6E). As for *GhAKT2bD* in Cluster III, its transcripts gradually reduced after low K^+^ treatment and reached a fairly low level after 48 h of treatment (Figure 6H). The expression of *GhKAT3aD* in Cluster IV showed an increase and peaked at 24 h after K^+^ deficiency, then rapidly decreased to the original level after 48 h of treatment (Figure 6I).

**Figure 7:**
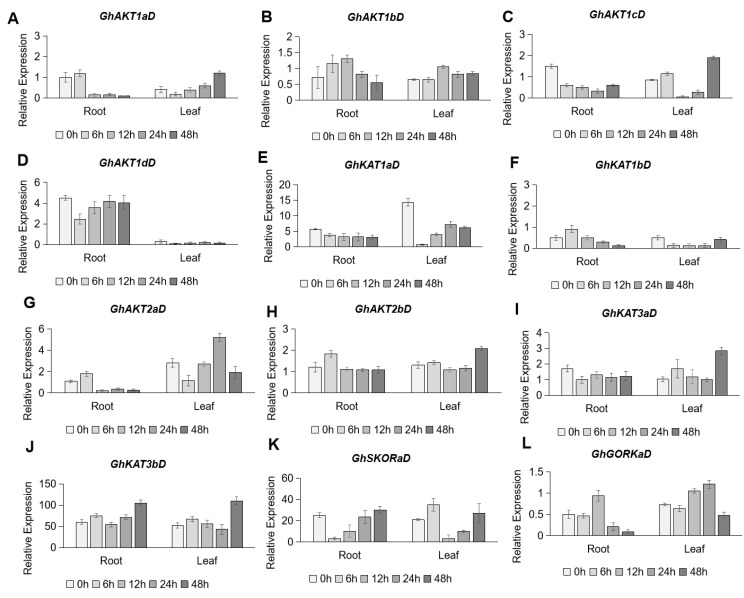
Time-course expression profiling of *Shaker* K^+^ channel family genes from the D subgenome in response to salt stress (250 mM NaCl). (**A**–**L**) indicate *GhAKT1aD* (**A**), *GhAKT1bD* (**B**), *GhAKT1cD* (**C**), *GhAKT1dD* (**D**), *GhKAT1aD* (**E**), *GhKAT1bD* (**F**), *GhAKT2aD* (**G**), *GhAKT2bD* (**H**), *GhKAT3aD* (**I**), *GhKAT3bD* (**J**), *GhSKORaD* (**K**) and *GhGORKaD* (**L**), respectively.

Under salt stress (250 mM NaCl), the expression of *Shaker* K^+^ channel family genes showed various responses (Figure 7). In the roots, the expression levels of *GhAKT1cD* (Figure 7C) in Cluster I, *GhKAT1aD* (Figure 7E) in Cluster II, and *GhKAT3aD* (Figure 7I) in Cluster IV showed a continuous downward trend under salt stress. However, the expression of *GhAKT1aD* (Figure 7A) and *GhAKT1bD* (Figure 7B) in Cluster I, *GhKAT1bD* (Figure 7F) in Cluster II, *GhAKT2aD* (Figure 7G) and *GhAKT2bD* (Figure 7H) in Cluster III and *GhGORKaD* (Figure 7L) in Cluster V showed a slight increase within 12 h after salt stress and then decreased to a different extent. In addition, it was found that salt stress rapidly (within 6 h) reduced the expression of *GhAKT1dD* (Figure 7D) and *GhSKORaD* (Figure 7K), and then their transcripts gradually recovered to the original level after 48 h of treatment. Among the 12 *Shaker* K^+^ channel family genes in roots, only the expression of *GhKAT3bD* in Cluster IV maintained a gradual increase in response to salt stress (Figure 7J). In the leaves, the expression of *GhAKT1aD* (Figure 7A) in Cluster I showed a roughly gradual increase under salt stress, and that of *GhAKT2bD* (Figure 7H), *GhKAT3aD* (Figure 7I) and *GhKAT3bD* (Figure 7J) did not increase until 48 h of salt treatment. Moreover, *GhAKT1dD* (Figure 7D), *GhKAT1aD* (Figure 7E) and *GhKAT1bD* (Figure 7F) were down-regulated overall by salt stress in leaves. Regarding other genes, no regular responses to salt were observed.

**Figure 8:**
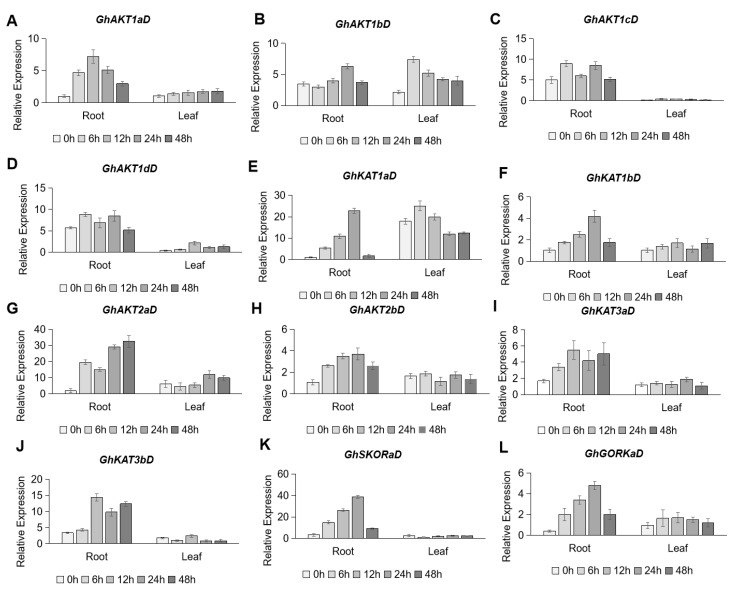
Time-course expression profiling of *Shaker* K^+^ channel family genes from the D subgenome in response to dehydration stress (15% PEG6000). (**A**–**L**) indicate *GhAKT1aD* (**A**), *GhAKT1bD* (**B**), *GhAKT1cD* (**C**), *GhAKT1dD* (**D**), *GhKAT1aD* (**E**), *GhKAT1bD* (**F**), *GhAKT2aD* (**G**), *GhAKT2bD* (**H**), *GhKAT3aD* (**I**), *GhKAT3bD* (**J**), *GhSKORaD* (**K**) and *GhGORKaD* (**L**), respectively.

Under dehydration stress (15% PEG6000), all *Shaker* K^+^ channel genes from the D subgenome were remarkably up-regulated in roots, with most of them being up-regulated quickly (within 6 h of treatment). Among them, the expression of *GhAKT1aD* (Figure 8A) peaked at 12 h of treatment, and that of *GhKAT1aD* (Figure 8E), *GhKAT1bD* (Figure 8F), *GhSKORaD* (Figure 8K) and *GhGORKaD* (Figure 8L) obviously peaked at 24 h of treatment. The two *GhAKT2s* (Figure 8G,H) and two *GhKAT3s* (Figure 8I,J) still showed a higher expression level 48 h after treatment. These results indicate that cotton *Shaker* K^+^ channels may play important roles in the regulation of draught stress. In leaves, the expression level of *Shaker* genes varied slightly except for *GhAKT1bD* (Figure 8B) and *GhKAT1aD* (Figure 8E), which were induced by dehydration at 6 h of treatment, then gradually declined (Figure 8).

### 3.7. Characterization and Function of the Cotton Shaker K^+^ Channel GhKAT1aD

Previous studies have revealed that KAT1 and KAT1-like channels can regulate plant stomatal movement [28,29,30,31]. However, the KAT1-like channel in cotton has not been reported yet. Therefore, we selected *GhKAT1aD* from the cotton *Shaker* K^+^ channel family genes to investigate its characterization and function.

**Figure 9:**
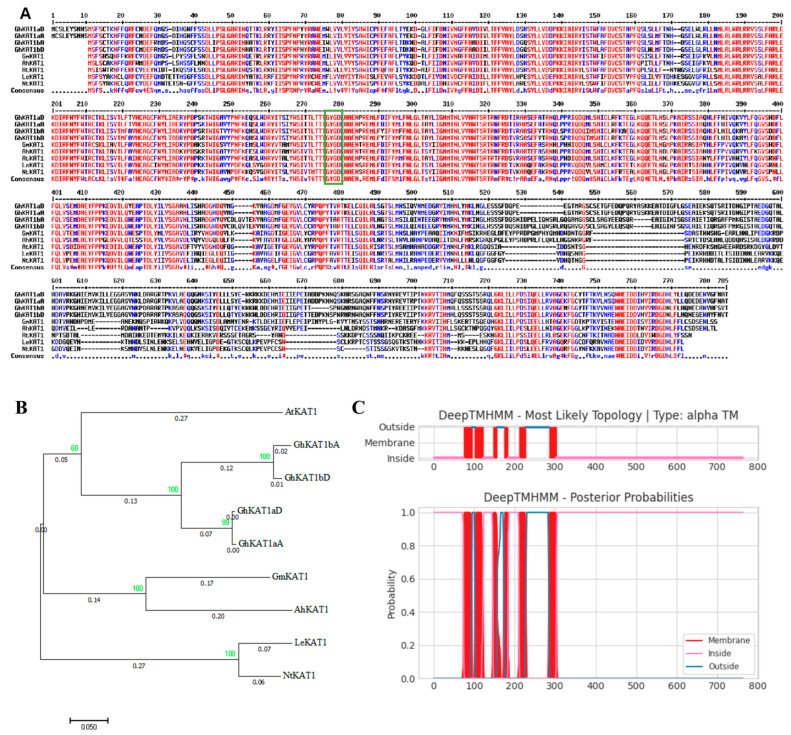
Characteristics and activity of the GhKAT1aD protein. (**A**) The protein sequence alignment of KAT1 in different species, including cotton (*Gossypium hirsutum*), soybean (*Glycine max*), peanut (*Arachis hypogaea*), Arabidopsis (*Arabidopsis thaliana*), tomato (*Lycopersicon esculentum*) and tobacco (*Nicotiana tabacum*). The green box contains ‘GYGD’ conserved sequence. (**B**) Phylogenetic tree of KAT1 in the above species. (**C**) Transmembrane structure prediction of GhKAT1aD.

The protein sequence alignment of cotton GhKAT1s, soybean (*Glycine max*) GmKAT1, peanut (*Arachis hypogaea*) AhKAT1, *Arabidopsis* AtKAT1, tomato (*Lycopersicon esculentum*) LeKAT1 and tobacco (*Nicotiana tabacum*) NtKAT1 were shown by using HMM (bootstrap ≥ 60). It is clear that all these predicted shaker proteins have a typical “GYGD” domain, which is a symbol of shaker K^+^ channel gene family members (Figure 9A). As shown in the phylogenetic tree (Figure 9B), GhKAT1s has the closest similarity to AtKAT1 of *Arabidopsis*. Moreover, GhKAT1aD is predicted to be a transmembrane protein containing six transmembrane segments (Figure 9C), which is consistent with the characteristics of the transmembrane structure of the *shaker* K^+^ channel gene family.

**Figure 10:**
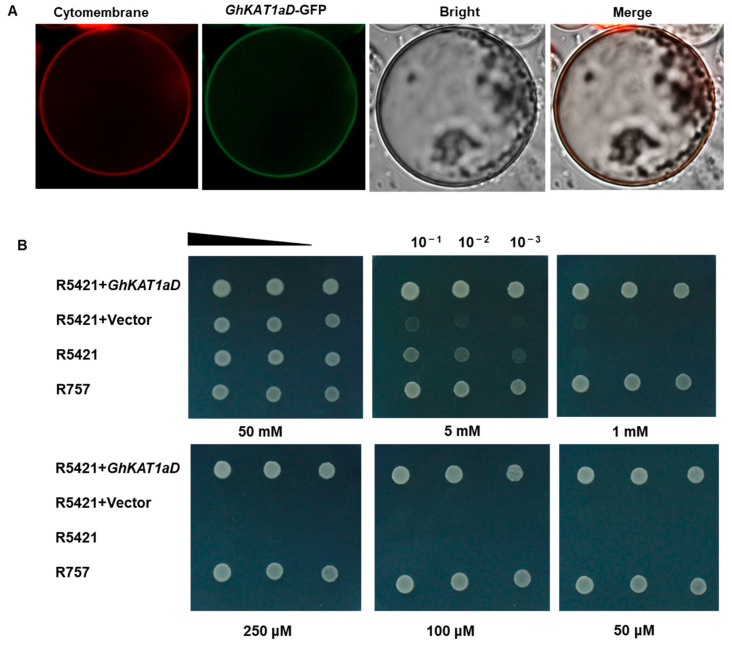
Subcellular localization of GhKAT1aD in plants (**A**). Transient expression of p35s::GhKAT1aD-GFP and plasma membrane marker p35S::PIP2-RFP in protoplasts isolated from cotton cotyledons (3 d after full expansion). Signals were monitored using confocal microscopy. GhKAT1aD restored the growth of yeast mutant R5421 defective in K^+^ uptake (**B**). GhKAT1aD was constructed into the p416-GPD vector and transformed into the yeast strain R5421. Strain R757 was used as a positive control. Yeasts were cultured at different K^+^ concentrations at 30 °C for 2 d.

To determine the subcellular localization of GhKAT1aD, GhKAT1aD-GFP with green fluorescence and a plasma membrane marker (p35S::PIP2-RFP) with red fluorescence were generated. They were transferred together into protoplasts isolated from cotton cotyledons. As shown in Figure 10A, the fluorescence signals derived from the GhKAT1aD-GFP construct were observed in the plasma membrane, which revealed that GhKAT1aD is a membrane-localized protein.

To examine the function of *GhKAT1aD*, the assay of the growth of yeast mutant R5421 (*trk1*∆, *trk2*∆), defective in K^+^ uptake, was employed. There were no differences in growth between R5421 and its wild-type counterpart, R757, under high K^+^ conditions (50 mM K^+^). However, the growth of R5421 was considerably depressed with the reduction of K^+^ concentration in the medium. Interestingly, the expression of *GhKAT1aD* in the R5421 strain completely restored its growth, even at 50 μM K^+^ supply (Figure 10B), suggesting that *GhKAT1aD* has the activity of K^+^ transport.

**Figure 11:**
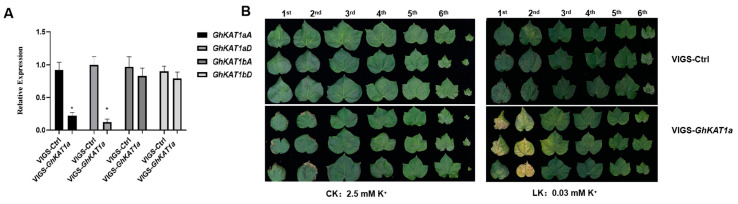
GhKAT1a positively regulates cotton growth under low K^+^ stress (LK, 30 μM). Silencing of *GhKAT1s* in cotton variety R15 by VIGS (agrobacterium-mediated virus-induced gene silencing) was conducted when the cotyledons were fully expanded. Fourteen days later, VIGS-*GLA1* plants (*cloroplastos alterados 1*, a marker to monitor silencing reliability) showed an albino phenotype, and leaf samples were collected to detect the expression of *GhKAT1a* (**A**). The expression of *GhKAT1aD* in VIGS-Ctrl leaves was regarded as ‘1′. *GhActin9* was used as the internal control. Then, VIGS-*GhKAT1a* plants and VIGS-Ctrl were transferred into sufficient (2.5 mM; CK) or low K^+^ (30 μM; LK) solutions for 33 d. The phenotype of plants (**B**) was photographed. The data are shown as means ± SD from three replicates (*n* = 3, * *p* ≤ 0.05).

**Figure 12:**
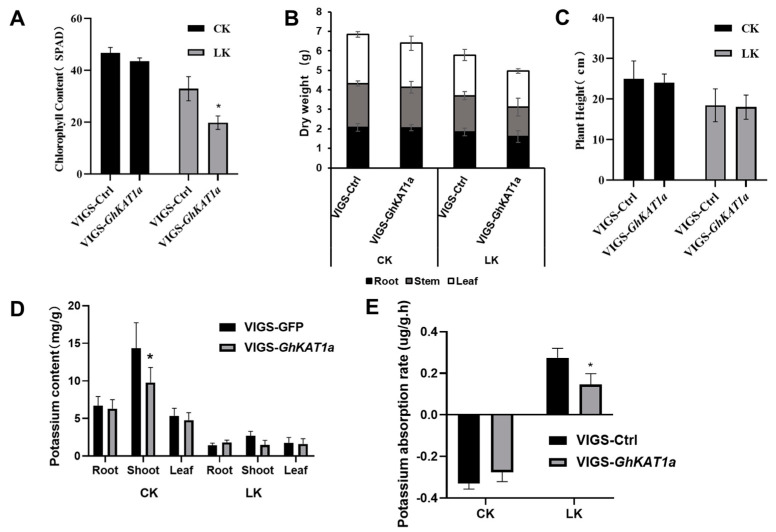
The comparisons between VIGS-Ctrl and VIGS-GhKAT1a in leaf SPAD value (average of the first, second and third leaf) (**A**), dry mass of roots, stem and leaves (**B**), plant height (**C**), K^+^ content in root, shoot, and leaf (**D**) and the net K^+^ uptake rate at the three-leaf stage (**E**). The data are shown as means ± SD from three replicates (*n* = 3, * *p* ≤ 0.05).

In addition, *GhKAT1a* in cotton variety R15 was silenced using the VIGS assay, and the differences in K^+^ nutrition between VIGS-*GhKAT1a* and VIGS-Ctrl were compared. The results showed that the expression of *GhKAT1aD* and *GhKAT1aA* decreased by 88% and 74% in VIGS-*GhKAT1a* plants, respectively (Figure 11A), indicating efficient silencing. The VIGS-*GhKAT1a* plants with six true leaves did not differ with VIGS-Ctrl plants in leaf color (Figure 11B), leaf SPAD value (average of the first, second and third leaves) (Figure 12A), biomass (Figure 12B) and plant height (Figure 12C) while grown in K^+^-sufficient (2.5 mM) solutions, whereas they displayed more severe K^+^ deficiency symptoms in the first and second leaves (Figure 11B), lower leaf SPAD value (Figure 12A) and less biomass (Figure 12B) under low K^+^ stress (30 μM). Furthermore, the plants subjected to low K^+^ stress had a much lower K^+^ content in roots, stems and leaves, and the K^+^ content in stems of VIGS-*GhKAT1a* plants was considerably lower than that of VIGS-Ctrl plants irrespective of the K^+^ supply levels (Figure 12D). Lastly, the net K^+^ uptake rate was investigated. The plants grown under K^+^ sufficiency (2.5 mM) showed a negative K^+^ uptake rate while the initial K^+^ concentration of the measurement solution was 0.1 mM, and the silencing of *GhKAT1a* did not affect the K^+^ uptake. However, those plants deprived of K^+^ had a positive net K^+^ uptake rate, and the VIGS-*GhKAT1a* plants showed a significant 48.1% lower K^+^ uptake rate than the VIGS-Ctrl plants (Figure 12E).

## 4. Discussion

### 4.1. Possible Functions of Shaker K^+^ Channel Family Genes in Roots, Leaves and Fiber Development

#### 4.1.1. Root

*AKT1* was the earliest reported *Shaker* K^+^ channel in plants; it is expressed in the epidermis and cortex cells of *Arabidopsis* root [32]. This gene encoded an inwardly rectifying K^+^ channel that mediated K^+^ uptake at conditions as low as 10 μM K^+^ [33]. *Arabidopsis akt1* mutant exhibits a reduced K^+^ absorption capacity in the roots, such that shoot yellowing and chlorosis occur compared with the wild type under low potassium stress. Previous work has suggested that *GhAKT1bD* is predominantly located in cotton leaves with low abundance in the roots, stem and shoot apex, and it may play a certain role in the K^+^ transport and distribution in leaves while facilitating K^+^ uptake in the roots [23]. In this study, Cluster I covered eight *GhAKT1s* that are also expressed constitutively. Furthermore, *GhAKT1cs* and *GhAKT1ds* showed higher expression in roots compared with *GhAKT1bs*, suggesting that they may play more important roles in K^+^ acquisition.

Cluster IV covered four *GhKAT3s*. *AtKAT3* was expressed in roots, its knockout mutants varied considerably in gating characteristics and cation sensitivity, but still mediated inward K^+^ currents in root hair, thus suggesting that *AtKAT3* can serve as an integral part of the functional K^+^ uptake channel [34]. A relevant study has reported that the AtKAT3 subunit may heteropolymerize with all inwardly rectifying K^+^ channel subunits (AKT1, KAT1, KAT2 and AKT2) and play a similar role in preventing K^+^ loss [11]. In this study, the possible reason for the high expression of *GhKAT3* in the roots may be attributed to its ability to form heteropolymers with inward rectification channels (GhAKT1s) and play a role in the K^+^ absorption process in cotton roots.

#### 4.1.2. Leaves

Cluster II contains four *GhKAT1s. KAT1* is located in *Arabidopsis* guard cells and can control the opening and closing of stomata by mediating K^+^ influx in guard cells [30,35,36]. The RNA-seq data and qRT-PCR analysis indicated that *GhKAT1* was highly expressed in the leaves, and we speculate that it can control the inflow of K^+^ and stomatal opening and closing in cotton leaves.

Cluster III is composed of four *GhAKT2s*. *AKT2* is mainly expressed in the phloem of *Arabidopsis*, mediates the bidirectional flow of K^+^, and realizes the loading and unloading of K^+^ in the phloem [37,38]. Zhang et al. (2022) characterized four GhAKT2 K^+^ channels from cotton and suggested that xylem- and phloem-expressed *GhAKT2bD* can facilitate K^+^ allocation [24]. In this study, the *GhAKT2s* also achieved constitutive expression, indicating their crucial roles in the growth and development of cotton plants.

#### 4.1.3. Fiber Development

Potassium is the most abundant mineral element in mature fibers and provides the necessary expansion pressure during fiber elongation [39]. In addition, K^+^ may play a certain role in the secondary wall formation of cotton fibers [40,41,42]. Field studies have suggested that an increase in fiber K^+^ concentrations does indeed yield longer fibers with higher quality [43]. During cotton fiber development, the high-affinity K^+^ uptake gene *GhKT1* was preferentially expressed at 10 DPA, driving the rapid phase of fiber elongation [44]. Moreover, the K^+^ transporter gene *Gh_D01G1760* (*HAK5*) is induced by low K^+^ in fibers cultured for 10 d [45].

As depicted in Figure 5, the *Shaker* K^+^ channel genes *GhAKT1bA*, *GhAKT2bA* and *GhAKT2bD* are highly expressed during fiber initiation and elongation, suggesting their roles in fiber development.

### 4.2. Possible Functions of the Shaker K^+^ Channel Gene Family in Stress Responses

Gene expression is correlated with gene function. There are a large number of defense and stress response elements in the promoter region of the *Shaker* K^+^ channel genes (Figure 4), suggesting that they are involved in the stress signaling pathway.

Salt stress is an abiotic stress correlated with modern agricultural production. There have been some reports that *Shaker* K^+^ channel genes can alleviate salt stress in plants. The results of Obata et al. (2007) indicated that Na^+^ decreased in yeast cells expressing *OsKAT1* as K^+^ increased under salt stress, and rice cells overexpressing *OsKAT1* had improved salt tolerance and increased cellular K^+^, suggesting that *OsKAT1* may play a certain role in the maintenance of cytosolic cation homeostasis and protect cells from Na^+^ during salt stress [46]. The expression of the AKT1-type K^+^ channel gene from *Puccinellia tenuiflora*, *PutAKT1*, can enhance salt tolerance in *Arabidopsis* [47]. In this study, the qRT-PCR results showed that the expressions of *GhKAT3bD* and *GhSKORaD* were up-regulated in roots, and *GhAKT1aD* and *GhAKT2bD* were up-regulated in leaves after 250 mM NaCl treatment (Figure 7), indicating that they may play a role in salt stress.

Huang et al. (2019) found that the overexpression of the *ClAKT* gene in chrysanthemum can improve drought tolerance [48]. In addition, the overexpression of *HvAKT1* improves barley drought tolerance by regulating root ion homeostasis and reactive oxygen species (ROS) and NO signaling [49]. In this study, the qRT-PCR analysis showed that all *Shaker* K^+^ channel genes from the D genome of cotton were considerably up-regulated in roots after osmotic stress (Figure 8), suggesting that the *Shaker* K^+^ channels may improve cotton drought tolerance.

### 4.3. Physiological Function of GhKAT1aD

KAT1 is a hyperpolarization-gated, inwardly rectifying K^+^ channel in plants and is mainly expressed in guard cells. Physiologically, it tunes the osmotic potential to hydraulically control stomatal opening in flowering plants [7]. In this study, *GhKAT1aD* from cotton leaves was cloned and its molecular characteristics and expression pattern investigated. The results showed that GhKAT1aD has the highest amino acid similarity (61.13%) with AtKAT1 (Figure 9B) and contains the conserved “GYGD” domain of *shaker* K^+^ channels (Figure 9A). Moreover, GhAKT1aD is predicted as a transmembrane protein containing six transmembrane segments (Figure 9C) and is localized at the plasma membrane (Figure 10A), all of which is consistent with the characteristics of the *shaker* K^+^ channel gene family [50,51].

According to the public RNA-Seq data, *GhKAT1aA* and *GhKAT1aD* were highly expressed in the stem and flowers. Additionally, their expression level was higher in the leaves than in the roots (Figure 5). In addition, *GhKAT1aD* in cotton leaves was rapidly (3 h) down-regulated by low K^+^ (30 μM) and NaCl (250 mM) stress, and then restored somewhat (Figure 6 and Figure 7). However, *GhKAT1aD* in cotton roots but not leaves was rapidly and sharply induced by dehydration (15% PEG6000) (Figure 8). Therefore, it was speculated that *GhKAT1a* may function in different cotton organs and be involved in various developmental and physiological processes as well as in response to a variety of stresses.

Furthermore, the expression of *GhKAT1aD* completely complemented the growth of a yeast mutant defective in K^+^ uptake even under the 50 μM K^+^ condition (Figure 10B), indicating that GhKAT1aD is capable of transporting K^+^. Additionally, the function of GhKAT1a through the VIGS assay was examined. The results showed that VIGS-*GhKAT1a* plants showed more severe symptoms of K^+^ deficiency (Figure 11B), suggesting that GhKAT1a probably participates in the responses of cotton to low K^+^ stress. Interestingly, the VIGS-*GhKAT1aD* plants grown in solutions with low K^+^ (30 μM) had a considerably lower (48.4%) net K^+^ uptake rate compared with VIGS-Ctrl plants (Figure 12E). We suppose that GhKAT1a may either engage in K^+^ uptake directly or affect K^+^ uptake indirectly via regulating stomatal opening. The latter means that the lower stomatal conductance of VIGS-*GhKAT1a* plants would reduce the transpiration rate and subsequently limit the transport of K^+^ from the roots to aboveground as well as the K^+^ uptake from the medium. Future work will reveal the exact mechanisms of the effect of *GhKAT1a* on the K^+^ uptake.

## 5. Conclusions

In this study, the 24 *Shaker* K^+^ channel genes were identified in cotton (*Gossypium hirsutum* L.), and they were classified into five clusters through a phylogenetic analysis. Subsequently, their chromosomal location, conserved motifs and gene structure were analyzed. The promoter region of cotton *Shaker* K^+^ channel genes covered a variety of cis-regulatory elements, including drought-, low-temperature-, phytohormone-response elements, etc. The transcriptome data showed that the *Shaker* K^+^ channels may play roles in root K^+^ uptake, shoot K^+^ transport and fiber development. Additionally, the *Shaker* K^+^ channels in cotton were probably involved in the regulation of drought stress. More importantly, one of the cotton *Shaker* K^+^ channel genes, *GhKAT1a*, may play a critical role in response to low K^+^ stress. Altogether, the current study broadened the understanding of K^+^ nutrition of cotton crops, and further studies will focus on the underlying mechanisms of K^+^ acquisition, transport and stress resistance performed by the *Shaker* K^+^ channel in cotton.

## Data Availability

No other data related to this study are available at this time.

## Supplementary Materials

The following supporting information can be downloaded at: https://www.mdpi.com/article/10.3390/life13071461/s1, Table S1: Primers for the characteristic of cotton *Shaker* K^+^ channel family genes.
