# Supplementary material for: Identification of Shaker Potassium Channel Family Members in Gossypium hirsutum L. and Characterization of GhKAT1aD
Source: Life (Basel). 2023 Jun 28;13(7):1461. doi: 10.3390/life13071461 (PMC10381577; doi:10.3390/life13071461)
Supplement: Supplementary file 1 [file life-13-01461-s001.zip › life-2326573-supplementary.pdf]

Table S1 Primers for the characteristic of cotton Shaker K^+ channel family genes

Primer	Sequence (5'→3')
	Primers for qRT-PCR
GhActin9-F	GCCTTGGACTATGAGCAGGA
GhActin9-R	AAGAGATGGCTGGAAGAGGA
GhAKT1aD-F	CGTTCATCATTGGACACCG
GhAKT1aD-R	TGGACGGGATTGGCTACAT
GhAKT1bD-F	GTTGCGTTCGTATGGCTTGTT
GhAKT1bD-R	CCAGGATCACGGTATCTCGC
GhAKT1cD-F	GAGAGAAAGCAGCCATTACAGC
GhAKT1cD-R	CTCCAATGGTGAAACCCAAG
GhAKT1dD-F	GTTTCGTGGCGTTTCTAATGAC
GhAKT1dD-R	TTGCCTCTCCAACAACCTG
GhKAT1aD-F	ATGTGTAGCCTTGAGTATTC
GhKAT1aD-R	ACAGATCCAGGCAGAGTAAA
GhKAT1bD-F	ATGTGATTTACAGGTTACG
GhKAT1bD-R	TTCCTCCCATTGCTCCTTGC
GhAKT2aD-F	TCCAAGGGTGGGCATCTAT
GhAKT2aD-R	CTGCTTCATCTGTTACAATGGC
GhAKT2bD-F	GGCGTTTCTCAGTTCATCG
GhAKT2bD-R	CCCACTCTGCTATTGCCAGT
GhKAT3aD-F	GCCCAACACCTATTCTGGAA
GhKAT3aD-R	ACTGCTCCGTCCCATTCTT
GhKAT3bD-F	GCCACAGCCATACACAGTTAGG
GhKAT3bD-R	TCATTGTTCCTTCCTCGGG
GhSKORaD-F	CAATGGTGTGGATAGATTACGG
GhSKORaD-R	TCTCTGAAGTCCTCAACCTCG
GhGORKaD-F	CCGAACACCACTTCATGTTG
GhGORKaD-R	TTCTCCCCTCGAATTTGTTG
	Primers for VIGS vectors

PYL156-*GhKAT1aD*-F	CGGGGTACCGTGGAACTTCACTGATGAAC
PYL156-*GhKAT1aD*-R	TCCCCCGGGAACACTTTTGGTGTCCTTCC
Primers for yeast complementation vectors	
p416-GPD-*GhKAT1aD*-F	CTTAGTTTCGACGGATTCTAGAATGTGTAGCCTTGAGTATTC
p416-GPD-*GhKAT1aD*-R	ATATCGAATTCCTGCAGCCCGGGGGTCGCATTGAAACCTACA
Primers for subcellular localization protein expression vector	
pHBT-*GhKAT1aD*-F	CTAGTCTAGAATGTGTAGCCTTGAGTATTC
pHBT-*GhKAT1aD*-R	TCCCCCGGGGGTCGCATTGAAACCTACATT
